# Risk of not being in employment, education or training (NEET) in late adolescence is signalled by school readiness measures at 4–5 years

**DOI:** 10.1186/s12889-024-18851-w

**Published:** 2024-05-22

**Authors:** Matthew Warburton, Megan L. Wood, Kuldeep Sohal, John Wright, Mark Mon-Williams, Amy L. Atkinson

**Affiliations:** 1 School of Psychology, University of Leeds, Leeds, LS2 9JT UK; 2 Bradford Institute for Health Research, Bradford, BD9 6RJ UK; 3 National Centre for Optics, Vision and Eye Care, University of South-Eastern Norway, Hasbergs vei 36, Kongsberg, 3616 Norway; 4 Department of Psychology, Lancaster University, Lancaster, LA1 4YF UK

**Keywords:** School readiness, Not in employment, education, or training, NEET, EYFSP, Academic attainment

## Abstract

**Background:**

Not being in employment, education, or training (NEET) is associated with poor health (physical and mental) and social exclusion. We investigated whether England’s statutory school readiness measure conducted at 4–5 years provides a risk signal for NEET in late adolescence.

**Methods:**

We identified 8,118 individuals with school readiness measures at 4–5 years and NEET records at 16–17 years using Connected Bradford, a bank of linked routinely collected datasets. Children were categorised as ‘school ready’ if they reached a ‘Good Level of Development’ on the Early Years Foundation Stage Profile. We used probit regression and structural equation modelling to investigate the relationship between school readiness and NEET status and whether it primarily relates to academic attainment.

**Results:**

School readiness was significantly associated with NEET status. A larger proportion of young people who were not school ready were later NEET (11%) compared to those who were school ready (4%). Most of this effect was attributable to shared relationships with academic attainment, but there was also a direct effect. Measures of deprivation and Special Educational Needs were also strong predictors of NEET status.

**Conclusions:**

NEET risk factors occur early in life. School readiness measures could be used as early indicators of risk, with interventions targeted to prevent the long-term physical and mental health problems associated with NEET, especially in disadvantaged areas. Primary schools are therefore well placed to be public health partners in early intervention strategies.

**Supplementary Information:**

The online version contains supplementary material available at 10.1186/s12889-024-18851-w.

## Background

NEET (not in employment, education, or training) is a major public health problem for many nations. It is expected that young people finishing compulsory education will move into further education, employment, or training, and those who do not do so are classified as being NEET. NEET is an administrative category used internationally to monitor the activity of young people and includes those often excluded from unemployment statistics (e.g., those who are not looking for employment). Here we focus on 16–17 year olds, but ages 16–24 years are also a focus in the UK [1], and other countries use various other age ranges [e.g. 2]. It is a useful indicator for increased risk of a range of negative long-term outcomes, including poor physical [3] and mental health [4], and social exclusion [5, 6]. NEET is more common in individuals from disadvantaged backgrounds [7, 8] and is seen as a major contributor to health inequity [9]. It follows that addressing NEET is key to ensuring good public health and social justice. NEET also has a wider societal and economic impact through loss of tax revenue and increased welfare payments [10].

Reducing the prevalence of those NEET has been a major area of policy development internationally, including the UK, France, and Germany [11]. Interventions are typically aimed at those already NEET [12], but identifying early indicators of NEET risk may allow more timely targeted intervention, thus addressing causes rather than symptoms. One of the largest predictors of NEET status in the UK is academic attainment at 15–16 years of age [13], just before individuals would usually be considered NEET. Earlier measures of academic attainment (at 10–11 years of age) also predict NEET status [8], propagating through later academic attainment [14].

In England, the Early Years Foundation Stage Profile captures a teacher-reported assessment of school readiness that measures a range of academic (e.g., literacy, mathematics) and non-academic abilities (e.g., personal, social, and emotional development) at 4–5 years of age. We have previously reported that children who fail to reach a Good Level of Development (GLD) on the profile had worse academic attainment at age 6–7 years [15], and identified children who are at increased risk of needing additional classroom support for special educational needs (SEN) [16]. Overall score on the Early Years Foundation Stage Profile has also been found to predict academic attainment at 15–16 years of age [17, 18]. Being “school ready” may, therefore, be associated with reduced likelihood of becoming NEET through its association with academic attainment. Furthermore, as the GLD indicator measures a range of academic and non-academic abilities, it may also relate to NEET status separately from any effect through academic routes.

We used the Connected Bradford dataset [19] to investigate the association between reaching a GLD and later NEET status. We first investigated whether reaching a GLD predicted later NEET status (at 16–17 years of age). We next examined whether achieving a GLD was associated with better performance at each intervening stage of academic assessment, and whether better attainment predicted lower NEET probability. We finally assessed whether the relationship between reaching a GLD and NEET was mainly realised through academic attainment.

## Methods

### Sample

The data were extracted from the Connected Bradford dataset, a linked database of over 800,000 citizens within the Bradford district [19]. Bradford, located in the north of England, is the fifth largest local authority by population, with a population of over 550,000 [20]. Bradford has a young population, with a median age four years below the national average (37 years in Bradford vs. 41 years in England), and a high level of diversity, with a large South Asian population. Bradford is the 5th most income deprived district in England, with 87 of the district’s Lower layer Super Output Areas (covering approximately 150,000 people mostly concentrated around central Bradford) being among the 10% most deprived in England [21]. Bradford trails the national average and neighbouring areas for percentage of children ‘school ready’ [22]. Bradford also has a higher rate of NEET in the 16–17 years age range compared to the national average [23].

Connected Bradford brings together a number of routinely collected data sources, including but not limited to primary and secondary health care, community care, social care, and education. Individuals are included in the database if they are registered at one of the 86 General Practitioners in the Bradford district, with additional datasets joined into this sample. Individuals in each dataset are referenced by a person ID (their pseudonymised NHS number) allowing data from different sources to be linked. The current study almost exclusively used the education dataset, which is well described in Sohal et al. (2022) [19].

The education dataset, provided by the Department for Education, is a reduced version of the National Pupil Database and contains tables relating to distinct items of information, for example academic performance at each Key Stage of schooling, the school census (collected three times per year), information on NEET status for those above 16 years of age, etc. We identified individuals within the education records who had data for both school readiness and NEET status. Because of the large temporal gap between these two pieces of information and the current availability of education data within Connected Bradford (up to the end of the 2018/2019 academic year), a single cohort of 8,129 individuals was available for analysis (entering Reception year in the 2006/2007 academic year and with NEET records in the 2018/2019 academic year at ages 16–17 years). In this cohort, 11 were missing data for one or more of the covariates and were thus excluded from the analysis due to their small number (though we note the analyses do not change with their inclusion). This gave a total sample of 8,118 individuals.

### Measures

#### GLD

Information about the GLD was contained within the Early Years Foundation Stage Profile table of the education dataset. The GLD is a binary measure (reached, not reached) which reflects school readiness [24]. To reach a GLD (pre-2013 version), children must reach at least six points on each of the core scales assessing: (i) personal, social, and emotional development; (ii) communication, language, and literacy, as well as at least 78 points across all assessed scales (including those described, plus (iii) problem solving, reasoning, and numeracy; (iv) knowledge and understanding of the world; (v) physical development; (vi) creative development).

#### NEET

Information about NEET status was contained within the National Client Caseload Information System table of the education dataset. There is a statutory requirement for UK local authorities to monitor and provide information to the Department for Education on post-16 activities, typically during the two academic years following the end of secondary school (i.e., ages 16–18 years) but in some cases (e.g., in the case of a ‘special needs’ plan) up until their 25th birthday. Local authorities maintain a local database (Client Caseload Information System) to identify and manage young people with no active education or training placement. This information is provided to the Department for Education, with both monthly and annual returns. Everyone within the dataset had multiple entries (typically one per month) listing their current activity code, which described the nature of their current circumstances (e.g., in full-time education; on an apprenticeship; seeking employment, education or, training). Activity codes classed as representing periods of NEET were those used by the Department for Education [25]. The derived binary outcome variable indicated whether an individual had *ever* been recorded with an activity code relating to being NEET during the academic year following secondary school (i.e., ages 16–17 years).

#### Academic attainment variables

Children in England progress through several Key Stages of education (KS1-KS4). Standardised assessments are conducted at multiple points, including at KS1 (Standard Assessment Tests (SATs), ages 6–7 years), KS2 (SATs, ages 10–11 years), and KS4 (General Certificate of Secondary Education (GCSE), ages 15–16 years). In the present sample, the assessments in KS1 and KS2 were scored at Levels 1–6, where Levels 2 and 4 were the expected level students should reach at KS1 and KS2 respectively. In both cases, students were tested on Mathematics, Reading, and Writing. The dataset for each Key Stage either includes their grade; a note indicating they were working below the assessed level, in which case their score was recoded as a zero; or a code detailing why they were not assessed, in which case their grade was left missing. In KS4, students had control over the subjects studied, though it was expected they would reach a Level 2 qualification (equivalent to grade 4 or above) in at least five subjects including English and Mathematics. The dataset includes binary variables indicating whether students reached the expected level in English, Mathematics, and any five subjects overall (regardless of whether they were entered for them), and binary variables indicating whether students were entered for examination in English, Mathematics, and any five subjects overall. Where students were not entered, the relevant variable indicating whether students reached the expected level was recoded as being missing.

In total, 160 (2%) pupils had missing exam records at KS1, 695 (9%) at KS2, and 822 (10%) at KS4. At KS1, 153 of the total 160 missing entries were because pupils were not listed in the KS1 dataset at all. At KS2 the missingness was more complicated. Of the 695 pupils missing data, 161 were not in the KS2 dataset, with a further 32 who were listed but not assessed. The remaining missing observations were where the student had valid entry for some, but not all, of the exams. Of these, 311 did not have a grade of 4 or above on the valid entries, indicating missingness was more common for pupils performing poorly. At KS4, 194 of the 822 missing entries were because pupils were not listed in the KS4 dataset. Of the remaining missing data, the majority (*n* = 314) were because students were not entered for five subjects overall nor English or Mathematics. Where they were entered for some but not others, it was more common that they did not reach a Level 2 qualification in the ones they did take, again indicating missingness was more common for poorly performing students. The qualitative reason for not being entered for certain exams is unclear, with many conceivable possibilities that do not necessarily reflect their ability (e.g. illness, family circumstances), so we deemed it safest for the main analyses to leave these as missing entries. However, because the substantiative effect of failing an exam or not taking it at all are likely to be similar on future education or employment opportunities (where often there is a requirement for certain grades to have been met), we report additional analyses in Supplemental Materials that addressed whether coding unelected exams as missing or below expected influenced the results. To summarise, we found the same results as presented in the main text with practically identical coefficients, indicating the effect estimates are not influenced by this coding choice.

#### Control variables

For both the regression and structural equation models, we ran models controlling for the following covariates: sex, ethnicity, academic month of birth (relative to the start of the academic year in September), English as an additional language status, whether the student received support for SEN, and whether the student was eligible for free school means (as a proxy for socioeconomic disadvantage). These variables were retrieved either from primary and secondary care records or from the school census. These variables have previously been associated with performance on the GLD [26] and/or later NEET status [13] and their influence may change with time. For example, being born later in the academic year (so-called summer born children) has long been known to be associated with worse academic attainment [27] but the effect appears to decrease across Key Stages [28].

Sex and academic month of birth were attained from healthcare data (primary and secondary care records), with the former coded as a binary variable (reference level = female), and the latter coded as a continuous variable centred on September (i.e., September = 0, October = 1, etc.). As the ethnic demographic of Bradford is mostly White British and South Asian heritage (91% of the current sample), ethnicity was coded as a categorical variable with White British (reference level), South Asian (covering the Indian, Pakistani, and Bangladeshi categories from the 2011 census definitions), and Other as categories. Ethnicity was taken from healthcare data where available and supplemented with the modal entry from the school census (collected three times per academic year) to reduce missingness where required. Where entries from both the national and school censuses were available, there was good agreement (93%). English as an additional language was extracted from the school census data by creating a binary variable where the reported first language is equal to English or Other, and then taking the modal entry.

The school census had variables that indicated whether students were currently receiving any support for SEN (either put in place by the school or the local authority), and whether students were currently eligible for free school meals. We created variables that indicated whether the student ever received any SEN support or was ever eligible for free school meals between the start of KS1 and the end of KS4, as being disadvantaged at any point may affect the probability of becoming NEET (see [15] for a similar approach). Table 1 shows the sample demographics for the complete sample.

**Table 1:** Demographic information for all variables

	*N* (%)
**NEET**	
Never NEET	7432 (91.5%)
Ever NEET	686 (8.5%)
**GLD**	
*Not reached*	4856 (59.8%)
Reached	3262 (40.2%)
**Free school meals**	
*Never eligible*	4650 (57.3%)
Ever eligible	3468 (42.7%)
**Special educational needs**	
*Never received support*	4463 (55.0%)
Ever received support	3655 (45.0%)
**Sex**	
*Female*	3873 (47.7%)
Male	4245 (52.3%)
**English as additional language**	
*False*	5034 (62.0%)
True	3084 (38.0%)
**Ethnicity**	
*White British*	4392 (54.1%)
South Asian	3034 (37.4%)
Other	692 (8.5%)
**Academic month of birth – Mean (SD)**	5.5 (3.5)

Note: The variable levels in italics show the reference category used in the analyses for binary variables

### Statistical analyses

All statistical analyses were conducted in R (version 4.3).

**Fig. 1:**
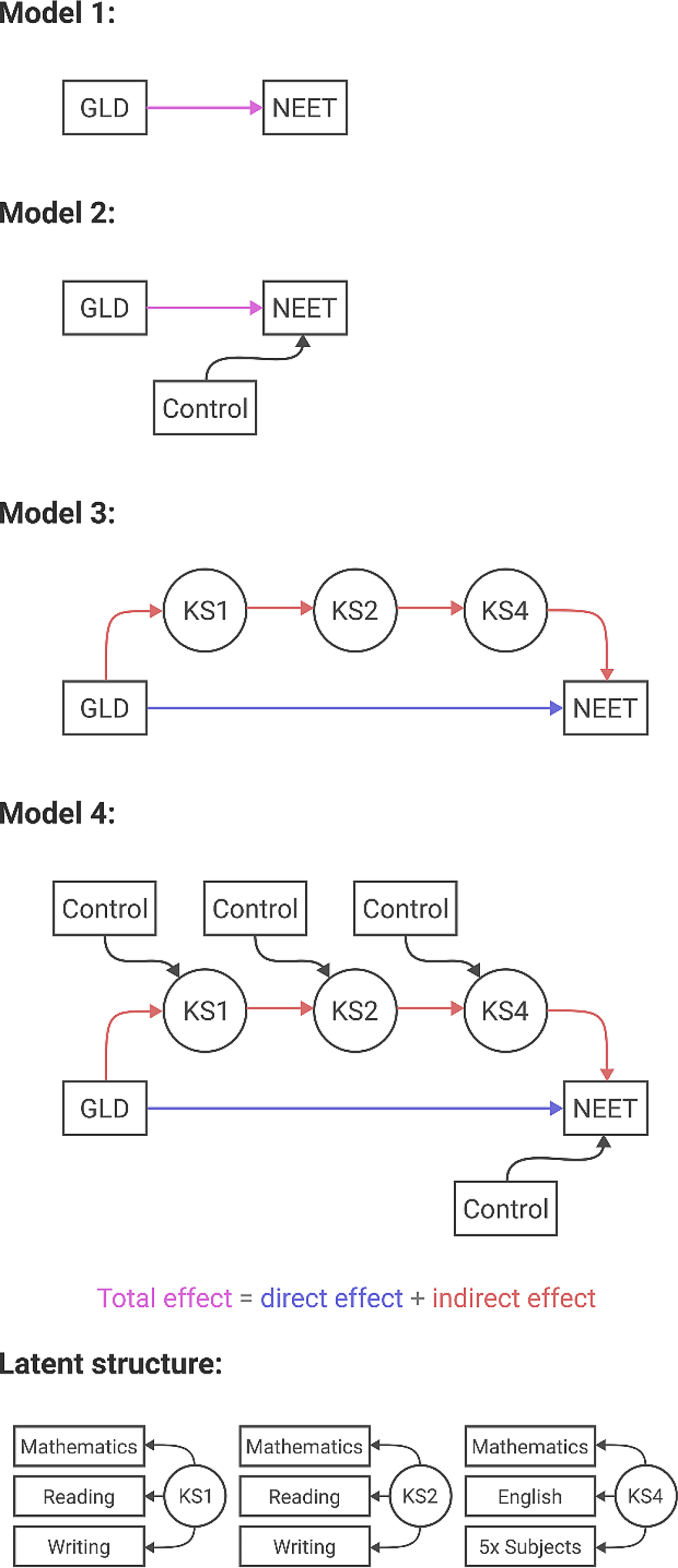
Graphical demonstration of the models fit to assess the relationship between GLD and NEET Note: In models 1 and 2, the total effect of GLD on NEET is assessed, whereas in models 3 and 4 the total effect is assessed through the direct effect of GLD on NEET, as well as its indirect effect through intervening academic attainment. In models 2 and 4, Control refers to the covariates (see text for a description of these variables). Latent variables were used to represent academic attainment at KS1, KS2, and KS4

Graphical representations of the models tested are shown in Fig. 1. We performed two probit regression analyses to investigate the overall association between GLD and NEET. For Model 1, the unadjusted regression, NEET was regressed solely on GLD, whereas Model 2 adjusted for covariates. Results of the regressions were reported both in terms of the coefficient estimates (in probits) and as average marginal effects on the probability scale (using the *margins* package). Further, to demonstrate the effect of being multiply disadvantaged, we used Model 2 to simulate the probability of becoming NEET with minimal or maximal disadvantage (i.e. those with none or all of the quantitatively negative effects on NEET respectively) [14], as well as showing the effect that failing or passing the GLD has in those scenarios respectively.

We next assessed whether GLD predicted later academic attainment, and whether academic attainment predicted NEET, to ensure there was evidence for reciprocal effects along an academic attainment pathway (see Supplemental Materials). Having found this, we then investigated whether the effect of GLD was associated with NEET status primarily through their association with academic attainment using structural equation modelling (using the *lavaan* package, the DWLS estimator, robust standard error estimates, and pairwise deletion for missing data). Latent variables representing underlying performance at each key stage were used. For KS1 and KS2, we include the grade achieved on Mathematics, Reading, and Writing separately as ordinal variables, and for KS4 we used binary variables for whether a student reached a Level 2 qualification in Mathematics, English, and any five subjects overall separately. While whether students reached the expected level in any five subjects overall at KS4 was highly correlated with doing so in English and Maths, for most students it was not dependent on them, so we used all three variables as indicators for the KS4 latent variable. For Model 3, the unadjusted model, we investigated the direct effect of GLD on NEET as well as its indirect effect through attainment on assessments at KS1, KS2, and KS4. The indirect effect estimate is calculated as the product of each path shown in red in model 3 in Fig. 1, with its standard error and significance calculated in *lavaan*. In total, there were 6686 (82%) complete cases, but as the missing attainment variables were indicators for latent variables, they could be handled with pairwise deletion. For Model 4, we controlled for the covariates at each time step (i.e., the regressions on KS1, KS2, KS4, and NEET). For all structural equation model analyses, we report the raw coefficient estimates (i.e., an identity link function for regressions on latent variables and a probit link function for regressions on NEET status).

## Results

### Is GLD associated with NEET status?

In total, 8.5% of the sample were ever NEET (Table 1) during the 2018/2019 academic year. 4% (*n* = 140) of those who achieved a GLD (*n* = 3,262) later went on to become NEET, whereas this was 11% (*n* = 546) for those who did not reach a GLD (*n* = 4,856). This is formalised in the unadjusted model (Model 1), where children who did not reach a GLD were significantly more likely to later be classified as NEET (Table 2). This relationship held after adjusting for covariates (Model 2, see Table 2). Several covariates were also significant predictors of later NEET status. Those who had ever received SEN support or were ever eligible for free school meals were significantly more likely to be NEET, whereas those who were born later in the academic year were less likely to become NEET. This model was then used to simulate the probability of becoming NEET for both the most advantaged and most disadvantaged person (one who has none or all the quantitatively negative effects of becoming NEET, respectively). For the most advantaged person, their probability of becoming NEET was estimated to be 1%, which increased to 3% if they failed to reach a GLD. In contrast, the most disadvantaged person had an estimated 25% probability of becoming NEET, which reduced to 17% if they reached a GLD.

**Table 2:** Parameter estimates for regression analyses of GLD attainment upon NEET status

	Model 1 (Unadjusted)			Model 2 (Adjusted)		
	Probits	SE	AME	Probits	SE	AME
Intercept	-1.21***	(0.02)	-	-1.66***	(0.07)	-
GLD – Reached	-0.50***	(0.05)	-0.07	-0.29***	(0.05)	-0.04
Ever eligible for free school meals				0.54***	(0.04)	0.08
Ever received SEN support				0.37***	(0.05)	0.05
Male				0.06	(0.04)	0.01
Ethnicity – South Asian				0.01	(0.08)	< 0.01
Ethnicity – Other				0.15	(0.08)	0.02
English as an additional language				-0.11	(0.08)	-0.02
Academic month of birth				-0.02***	(0.01)	<-0.01
N	8,118			8,118		
McFadden’s adjusted R^2^	0.03			0.09		
McKelvey and Zovoina’s R^2^	0.06			0.16		

Note: Coefficients are reported as probits. For continuous variables, the coefficient represents the change in outcome for a 1 unit change in the variable, whereas for binary variables the coefficient represents the change in the outcome when going from the reference category to another level (i.e. English as primary language → English as additional language). Significance is represented by asterisks: * Significant at *p* < .05 level, ** Significant at *p* < .01 level, *** Significant at *p* < .001 level. SE: Standard error, AME: Average marginal effect (probability scale)

### Is the GLD associated with NEET indirectly through academic attainment?

Additional analyses, to establish a possible academic pathway for the association, found that attaining a GLD predicted academic outcomes at KS1-4, and performance at KS1-4 predicted later NEET status (see Supplemental Materials). We therefore formally tested whether the association between GLD and NEET was realised indirectly through academic attainment.

In the unadjusted structural equation model analysis (Model 3, see Table 3), we found a strong association between achieving a GLD and performance at KS1, and evidence that academic performance at one time point positively predicts subsequent performance. Finally, improved academic performance at KS4 was associated with a reduced probability of becoming NEET. The *indirect* effect of GLD on NEET, acting through these academic paths (i.e., multiplying the regression coefficients for the paths represented in red in Fig. 1), made up the majority of the total effect seen in Model 1 (around 65%), but a significant *direct* effect was also observed.

In the adjusted structural equation model analysis controlling for covariates (Model 4, see Table 3), both the direct and indirect effects of GLD upon NEET remained significant. Several control variables were also significantly associated with the outcomes. Ever receiving SEN support or ever being eligible for free school meals was associated with worse performance at KS1 and KS4, as well as a higher likelihood of being NEET. The bulk of the effect of being eligible for free school meals appears to impact NEET status directly, whereas the bulk of the effect of SEN appears to pass through academic attainment (when comparing the effect of the variables on NEET between Tables 2 and 3). Increased academic month of birth had a detrimental effect upon KS1 performance but was associated with protective effects upon KS2 and KS4 performance, and on later NEET status. Being male and having English as an additional language had varying effects at different points in the academic journey. Finally, ethnicity was significantly associated with KS2 performance, where those from South Asian or Other ethnic groups performed better than White British peers, and with NEET status, where Other ethnic groups were more likely to become NEET. The results of these analyses hold when additional controls for missing data among the academic attainment measures are performed (see Supplemental Materials).

**Table 3:** Parameter estimates for the mediation analyses

Outcome	Predictor	Model 3 (Unadjusted)		Model 4 (Adjusted)	
		Coefficient	SE	Coefficient	SE
KS1	GLD – Reached	1.31***	(0.03)	0.94***	(0.03)
	Ever eligible for free school meals			-0.25***	(0.03)
	Ever received SEN support			-1.19***	(0.03)
	Male			-0.04	(0.03)
	Ethnicity – South Asian			0.02	(0.05)
	Ethnicity – Other			-0.04	(0.05)
	English as an additional language			-0.23***	(0.05)
	Academic month of birth			-0.03***	(< 0.01)
KS2	KS1	0.79***	(0.01)	0.75***	(0.01)
	Ever eligible for free school meals			0.00	(0.02)
	Ever received SEN support			-0.01	(0.03)
	Male			0.13***	(0.02)
	Ethnicity – South Asian			0.13**	(0.04)
	Ethnicity – Other			0.11**	(0.04)
	English as an additional language			0.07	(0.04)
	Academic month of birth			0.02***	(< 0.01)
KS4	KS2	0.83***	(0.01)	0.77***	(0.01)
	Ever eligible for free school meals			-0.27***	(0.03)
	Ever received SEN support			-0.13***	(0.03)
	Male			-0.16***	(0.03)
	Ethnicity – South Asian			0.05	(0.06)
	Ethnicity – Other			0.07	(0.05)
	English as an additional language			0.14**	(0.05)
	Academic month of birth			0.02***	(< 0.01)
NEET	GLD – Reached	-0.17***	(0.05)	-0.12*	(0.06)
	KS4	-0.39***	(0.02)	-0.31***	(0.03)
	Ever eligible for free school meals			0.41***	(0.05)
	Ever received SEN support			0.11*	(0.05)
	Male			0.03	(0.04)
	Ethnicity – South Asian			0.06	(0.09)
	Ethnicity – Other			0.19*	(0.08)
	English as an additional language			-0.09	(0.09)
	Academic month of birth			-0.02**	(0.01)
*NEET*	*GLD (Direct)*	-0.17***	*(0.05)*	-0.12*	*(0.06)*
*NEET*	*GLD (Indirect)*	-0.33***	*(0.02)*	-0.17***	*(0.02)*
n		8,118		8,118	
${\chi }^{2}$ / df		1,096 / 41		1,515 / 83	
*p*		< 0.001		< 0.001	
CFI		0.996		0.991	
RMSEA		0.056		0.046	

Note: Unstandardised coefficient estimates are reported. For KS1-4 as the outcome, these coefficient estimates are through the identity link function, whereas for coefficients on NEET these are through the probit link function. For continuous variables, the coefficient represents the change in outcome for a 1 unit change in the variable, whereas for binary variables the coefficient represents the change in the outcome when going from the reference level to another level (i.e. English as primary language → English as additional language). Significance is represented by asterisks: * Significant at *p* < .05 level, ** Significant at *p* < .01 level, *** Significant at *p* < .001 level. SE: Standard error, CFI: Comparative fit index, RMSEA: Root mean square error of approximation

## Discussion

The results show that performance on a school readiness assessment, conducted at age 4–5 years, was associated with NEET status at 16–17 years of age. Reaching a GLD was also predictive of academic attainment through primary and secondary school, and academic attainment at all time points was predictive of later NEET status. Investigating this further, we found that the overall effect of GLD on NEET was mainly realised indirectly through academic attainment, but a direct effect was also observed.

These findings indicate that data on school readiness, routinely collected by schools in England, could be used to identify individuals at increased risk of becoming NEET from an early age. Given that NEET status is strongly associated with a range of adverse health and social outcomes (e.g. mental [3] and physical health [4], social exclusion [5, 6]), identifying an early point for intervention has important implications for public health [9, 29]. This adds to a growing body of evidence that holistic measures of school readiness have predictive power for later outcomes [15–18], including similar measures collected in other countries [30, 31].

Given that school readiness measures aim to capture factors important for academic success, it is not surprising that the bulk of the relationship between the GLD and later NEET status is associated with academic attainment. One possibility is that academic abilities at school entry impact NEET status through a propagation of missing early academic building blocks. This is plausible, since more advanced concepts in the later school years rely heavily on core concepts taught earlier. It is also possible that in some cases a lack of school readiness indicates that a child is growing up in an environment that is not conducive to academic success. If the home environment contributed towards a lack of school readiness, then it is improbable that subsequently the home will provide a beneficial milieu for good academic attainment.

Further, the GLD additionally captures non-academic skills (e.g., social skills), and this may help explain the direct effect observed. It is possible that at least some of the non-academic aspects of school readiness may be important for NEET but less crucial for academic attainment. For example, whilst there is some debate concerning whether social skills are predictive of academic attainment [32, 33], these are greatly valued in the labour market [34]. Indeed, there is evidence that social skills in kindergarten are associated with employment outcomes in adulthood [35]. It would be useful if further research investigated *which* aspects of school readiness at school entry predict NEET status.

The current study’s design means it is not possible to causally implicate performance on the GLD with later NEET status. However, we believe it is unlikely that simply ‘training to test’ (where educators focus solely on improving performance on the assessment) would be effective. Instead, it is more likely that the GLD is reflecting broader developmental and environmental concerns that are related to later outcomes and need to be addressed in a holistic manner across public services. For example, the current analysis could not control for factors such as parental class and education (because of limitations of the data used) that may be captured by the school readiness measure. It would be beneficial for further research to use methods that get closer to a causal understanding of school readiness specifically, as well as examining effects on related outcomes such as physical and mental health [36].

Our results show that vulnerabilities compound, with deprivation (indexed by being eligible for free school meals) and SEN being associated with both academic attainment and NEET status. The probability of becoming NEET was estimated to be 1% for the most advantaged person, which increased to 3% if they failed to reach a GLD. In contrast, the most disadvantaged person had an estimated 25% probability of becoming NEET, which reduced to 17% if they reached a GLD. This identifies additional factors that are associated with NEET and suggests that early interventions may provide the most benefit by targeting children with multiple risk factors.

Other variables were also associated with NEET status. A later academic month of birth appeared to have a negative relationship with academic attainment at KS1 with evidence of catching up at KS2 and KS4, consistent with previous evidence [28], but had a protective effect upon NEET. The latter effect is unexpected, with previous work finding no effect of month of birth upon employment [37], so future work could investigate these different paths specifically. Finally, being in the “Other” ethnicity category had a direct association with a greater chance of being NEET, potentially pointing to structural inequalities faced by those from minority backgrounds. However, the category belies the diversity of ethnicities within, and future work using a larger sample may provide a better indication of such effects across smaller, more distinct ethnic groups.

A strength of this study is that it combines data from thousands of individuals across multiple life stages (the early years, primary and secondary school, post-secondary school). In doing so, it demonstrates the power that using routinely collected data could have to identify relationships between health, education, and other outcomes over an extended timeframe. Nevertheless, the current study does have several limitations. As the Early Years Foundation Stage Profile started to be widely used in 2007, the current data availability meant we were restricted to using NEET records only at 16–17 years of age, whereas NEET is typically considered over a longer time period (e.g. 16–24 years [1]). Further, the sample contains individuals in the Bradford district, which has a higher-than-average rate of NEET when compared to the rest of England [38]. These factors may limit generalisability of the results, though it is worth noting that being NEET at a young age is associated with worse long-term outcomes [39], so the observed relationship may be particularly important. Finally, we focussed on those who were ever NEET within our dataset, but the relationships observed may differ across sub-groups within this category. Future research could identify sub-groups based on factors like temporal patterns of NEET (e.g. short or long-term NEET periods) and investigate whether the associations differ.

## Conclusions

We demonstrate that a school readiness assessment conducted at 4–5 years of age identifies children who are nearly three times as likely to be NEET 12 years later (11% vs. 4%). This indicates the power of using routinely collected data to identify children at increased risk of becoming NEET. Given that NEET is associated with poor physical and mental health, these findings have important consequences for public health.

### Electronic supplementary material

Below is the link to the electronic supplementary material.

Supplementary Material 1

## Data Availability

The data that support the findings of this study are available through the Connected Bradford dataset, which can be accessed by bona fide researchers. The analysis scripts are freely available at: https://github.com/ConnectedBradford/CB_2116_NEET.

## Abbreviations

AME: Average marginal effect
GLD: Good level of development
KS: Key Stage
NEET: Not in employment, education, or training
SE: Standard error
SEN: Special educational needs
