# Supplementary material for: Risk of not being in employment, education or training (NEET) in late adolescence is signalled by school readiness measures at 4–5 years
Source: BMC Public Health. 2024 May 22;24:1375. doi: 10.1186/s12889-024-18851-w (PMC11110409; doi:10.1186/s12889-024-18851-w)
Supplement: Supplementary file 1 — Supplementary Material 1 [file 12889_2024_18851_MOESM1_ESM.pdf]

Supplementary Materials

Risk of not being in employment, education or training (NEET) in late adolescence is signalled by school readiness measures at 4-5 years

Matthew Warburton[1]*, Megan L. Wood[1], Kuldeep Sohal[2], John Wright[2], Mark Mon-Williams[1,2,3], Amy L. Atkinson[4]

[1]School of Psychology, University of Leeds, [2]Bradford Institute for Health Research, Bradford, West Yorkshire, UK, [3]National Centre for Optics, Vision and Eye Care, University of South-Eastern Norway, Kongsberg, Hasbergs vei 36, 3616, Norway, [4]Department of Psychology, Lancaster University

* Corresponding author: pscmwa@leeds.ac.uk

Contents

Relationships between GLD, academic attainment, and NEET 2

Structural equation model sensitivity analysis 4

Relationships between GLD, academic attainment, and NEET

Prior to the structural equation modelling analysis, we sought to confirm that GLD performance predicts later academic attainment, and that academic attainment predicts NEET status. See Supplementary Table 1 for the frequencies. We ran probit regressions examining whether reaching a GLD predicted whether students reached the expected levels at KS1, KS2, and KS4 overall (Levels 2 or 4 across all assessments at KS1 and KS2 respectively, and a Level 2 qualification on English, Mathematics, and at least 5 exams overall at KS4). Similarly, we investigated whether reaching expected levels at each academic stage predicted later NEET status in a similar trio of probit regressions. The KS1 regressions had 160 missing observations, KS2 had 695 missing observations, and KS4 had 822 missing observations, which were deleted row wise. We additionally ran versions controlling for the same covariates used in the main text.

Reaching a GLD increased one's probability of reaching expected levels at KS1 (β = 1.51 [1.43, 1.60], $p < .001$, AME = 37%), at KS2 (β = 1.04 [0.97, 1.10], $p < .001$, AME = 33%), and at KS4 (β = 0.88 [0.82, 0.94], $p < .001$, AME = 33%). These held when controlling for covariates: KS1 (β = 1.05 [0.94, 1.15], $p < .001$, AME = 21%), KS2 (β = 0.74 [0.66, 0.81], $p < .001$, AME = 22%), and KS4 (β = 0.62 [0.54, 0.69], $p < .001$, AME = 21%).

The probability of later becoming NEET was reduced when students reached expected levels at KS1 (β = -0.46 [-0.55, -0.38], $p < .001$, AME = -8%), KS2 (β = -0.45 [-0.53, -0.36], $p < .001$, AME = -7%), and KS4 (β = -0.75 [-0.86, -0.65], $p < .001$, AME = -8%), and this held when controlling for covariates: KS1 (β = -0.16 [-0.27, -0.06], $p = .002$, AME = -2%), KS2 (β = -0.25 [-0.35, -0.15], $p < .001$, AME = -3%), and KS4 (β = -0.65 [-0.77, -0.54], $p < .001$, AME = -7%).

Supplementary Table 1. The number of individuals who did or did not reach a Good Level of Development and performed below or at expected levels for the KS1-KS4 outcomes, and the number of individuals who performed below or at expected levels for the KS1-KS4 outcomes and were or were not NEET.

**Independent variable**		**Dependent variable**							
		KS1		KS2		KS4		NEET	
		Below expected	Expected	Below expected	Expected	Below expected	Expected	Not NEET	NEET
GLD	Not reached	1949	2813	1975	2307	2351	1849	..	..
	Reached	130	3066	404	2737	723	2373	..	..
KS1	Below expected	..	..	..	..	..	..	1782	297
	Expected	..	..	..	..	..	..	5509	370
KS2	Below expected	..	..	..	..	..	..	2086	293
	Expected	..	..	..	..	..	..	4771	273
KS4	Below expected	..	..	..	..	..	..	2752	322
	Expected	..	..	..	..	..	..	4128	94

Note: Total N within a pair of variables (e.g. GLD and KS1) will not add to the full sample due to missing academic observations.

Structural equation model sensitivity analysis

Because the majority of the missing values for academic attainment were not missing in the traditional sense, estimating them through techniques like multiple imputation seemed inappropriate. At least at KS4, not sitting or failing an exam are likely to have the same impact on future job prospects, as many jobs require a certain number of Level 2 qualifications, regardless of whether they were elected. We therefore conducted additional structural equation modelling analyses where missing values at KS2 and KS4 (other than those not in the dataset, described in the Methods section) were replaced with values indicating either the student reached a score of 0 at KS2 or the student did not reach a Level 2 qualification for the relevant exams at KS4.

In the unadjusted analysis (Model 3, missingness controlled: $\chi^2(41)$ = 689, p < .001, CFI = 0.997, RMSEA = 0.044), we found a strong association between achieving a GLD and performance at KS1 (β = 1.31 [1.26, 1.37], p < .001), and evidence that ability at one time point positively predicts subsequently ability (KS1 → KS2: β = 0.80 [0.79, 0.81], p < .001; KS2 → KS4: β = 0.60 [0.56, 0.64], p < .001). Finally, improved academic ability at KS4 is associated with a reduced probability of becoming NEET (β = -0.57 [-0.64, -0.51], p < .001). The *indirect* effect of GLD on NEET, acting through these academic paths, makes up the majority of the total effect seen in Model 1 (β = -0.36 [-0.40, -0.32], p < .001), but a significant *direct* effect also emerged (β = -0.14 [-0.24, -0.05], p = .004).

In the adjusted analysis controlling for covariates (Model 4, missingness controlled: $\chi^2(83)$ = 1507, p < .001, CFI = 0.991, RMSEA = 0.046), we found significant direct (β = -0.13 [-0.23, -0.02], p = .020) and indirect (β = -0.16 [-0.18, -0.14], p < .001) effects of GLD upon NEET.
